# Sphingolipid Metabolism: A New Therapeutic Opportunity for Brain Degenerative Disorders

**DOI:** 10.3389/fnins.2018.00249

**Published:** 2018-04-17

**Authors:** Alba Di Pardo, Vittorio Maglione

**Affiliations:** IRCCS Neuromed, Pozzilli, Italy

**Keywords:** neurodegenerative diseases, sphingolipid metabolism, S1P, ceramide, FTY720, S1PRs

## Abstract

Neurodegenerative diseases represent a class of fatal brain disorders for which the number of effective therapeutic options remains limited with only symptomatic treatment accessible. Multiple studies show that defects in sphingolipid pathways are shared among different brain disorders including neurodegenerative diseases and may contribute to their complex pathogenesis. In this mini review, we discuss the hypothesis that modulation of sphingolipid metabolism and their related signaling pathways may represent a potential therapeutic approach for those devastating conditions. The plausible “druggability” of sphingolipid pathways is greatly promising and represent a relevant feature that brings real advantage to the development of new therapeutic options for these conditions. Indeed, several molecules that selectively target sphingolipds are already available and many of them currently in clinical trial for human diseases. A deeper understanding of the “sphingolipid scenario” in neurodegenerative disorders would certainly enhance therapeutic perspectives for these conditions, by taking advantage from the already available molecules and by promoting the development of new ones.

## Introduction

**Figure 1:**
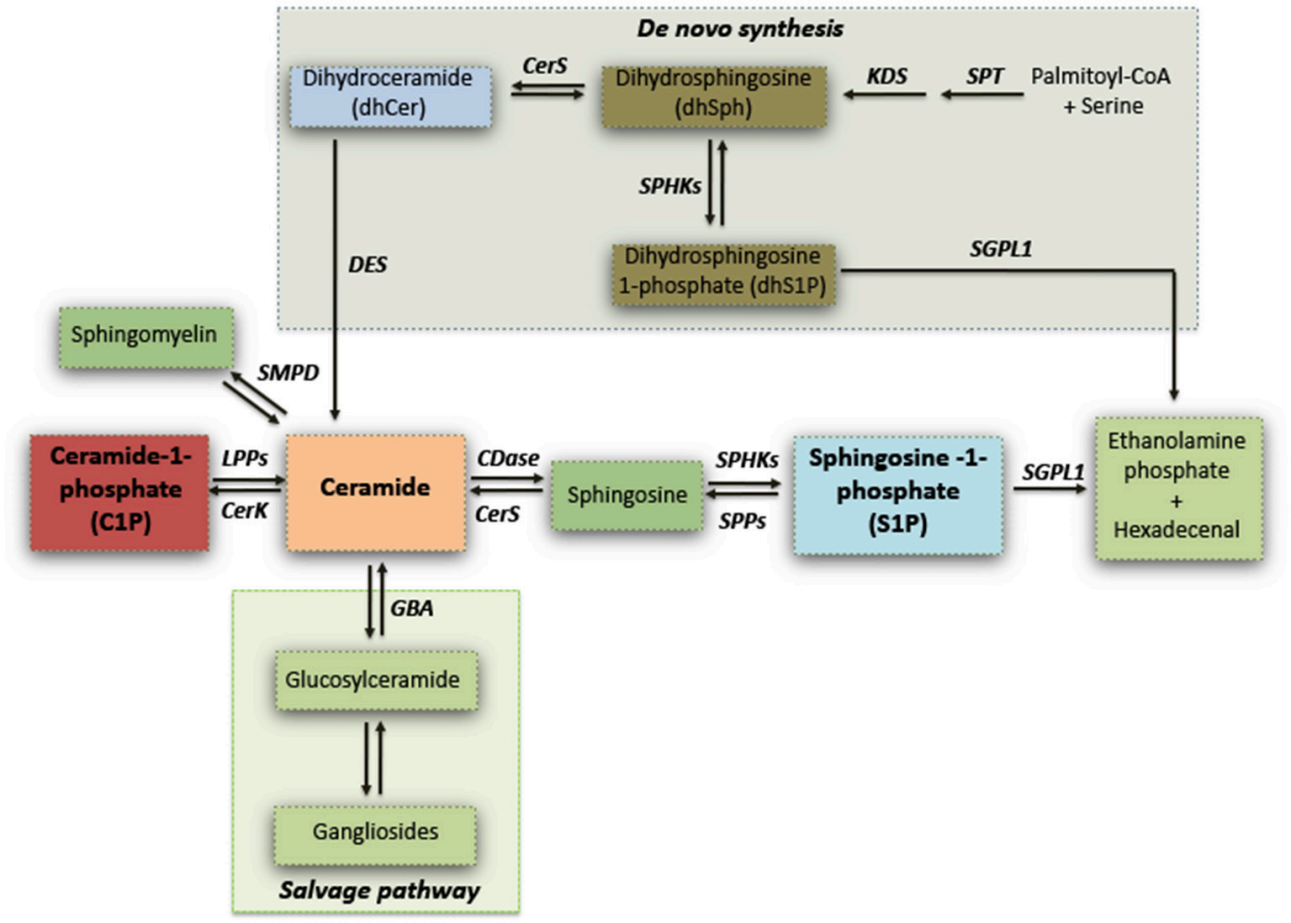
Simplified schematic representation of sphingolipid biosynthesis. Serine palmitoyltransferase (SPT) catalyzes the initial reaction of the *de novo* biosynthesis of sphingolipids. Dihydrosphingosine (dhSPH) is generated after an intermediate step by the action of 3-keto-dihydrosphingosine reductase (KDS). Successively, dhSph can be either phosphorylated, with the generation of dhSphingosine-1-phosphate by sphingosine kinases (SPHKs), or acetylated by ceramide synthase (CERS) and desaturated by ceramide desaturase (DES) to form ceramide. Ceramide may also derive from the *Salvage pathway* through either the hydrolysis of sphingomyelin or by the recycling of gangliosides by Sphingomyelin Phosphodiesterase (SMPD) and Glucosylceramidase (GBA) respectively. Ceramide can be phosphorylated by Ceramide kinase (CerK) with the generation of ceramide-1-phosphate (C1P) which in turn can be re-converted in ceramide by Lipid Phosphate Phosphatases (LPPs). Ceramide can be subsequently metabolized by Ceramidase (CDase) to generate sphingosine which, in turn, produces sphingosine-1-phosphate (S1P) through phosphorylation by SPHKs. S1P can be either dephosphorylated and re-converted to sphingosine by S1P Phosphatases (SPPs), or irreversible catabolized into hexadecenal + phospho-ethanolamine by S1P Lyase (SGPL1).

Sphingolipids have long been viewed as merely ubiquitous components of the cell membrane, and exert a critical role in regulating vital cell functions and formation of membrane microdomain “lipid rafts” for integrating cell signaling (Gault et al., 2010; Olsen and Faergeman, 2017). Sphingolipids synthesis can occur via *de novo* biosynthetic pathway or the hydrolysis of sphingomyelin, or can also derive by the “salvage pathway” which determines the recovery of sphingosine, by the recycling of complex sphingolipids (gangliosides) through a coordinated action of several enzymes (see Figure 1) (Gault et al., 2010).

Enzymatic pathways result in the formation of several different and tightly regulated lipid mediators such as ceramide, ceramide-1-phosphate (C1P), sphingosine, and sphingosine-1-phosphate (S1P) (Gault et al., 2010; Goñi et al., 2014).

Ceramide is pivotal in the synthesis of sphingolipids (Mullen et al., 2012). It benefits early growth and development of neuronal cells and, at low levels it promotes cell survival and division (Schwarz and Futerman, 1997; Brann et al., 1999). Defects in its metabolism may have deleterious effects, often resulting in its abnormal accumulation that causes an increase of the programmed cell death in different cell types including neurons (Tohyama et al., 1999; Jana et al., 2009; Mullen and Obeid, 2012; Czubowicz and Strosznajder, 2014).

Direct phosphorylation of ceramide by ceramide kinase (CerK) is the major identified mechanism for generation of C1P in cells (Bajjalieh et al., 1989). The role of C1P has been mainly studied in blood cells in which it regulates proliferation, migration, and survival (Hoeferlin et al., 2013). The function of C1P in the brain is still poorly investigated. However, the presence of CerK in synaptic vesicles (Bajjalieh et al., 1989), along with the evidence that C1P regulates photoreceptor homeostasis (Miranda et al., 2011) as well as P-glycoprotein transport at the blood-brain barrier (Mesev et al., 2017), strongly suggests that C1P may be involved also in the homeostasis of the central nervous system (CNS).

Similarly, S1P is a potent signaling molecule that, beside governing essential physiological processes like vascular, bone formation (Hla et al., 2008; Xiong and Hla, 2014; Holmes, 2015) and inflammatory response (Huang et al., 2013; Aoki et al., 2016), regulates many of the molecular events crucial for brain development and neuronal survival (Mendelson et al., 2014; van Echten-Deckert et al., 2014). S1P acts either in the intracellular or in the extracellular compartments (Strub et al., 2010; Mendelson et al., 2014). Intracellularly, S1P may play different roles depending on its subcellular localization and normally regulates mitochondria function (Strub et al., 2011; Shen et al., 2014), gene expression by inhibiting histone deacetylases (HDACs) (Hait et al., 2009; Riccio, 2010) and ER stress (Lépine et al., 2011; Park et al., 2016).

Outside the cell, S1P acts as a high affinity agonist at five known G protein-coupled receptors, S1PR_1_ -S1PR_5_, which in the brain are expressed by many CNS cell types (Blaho and Hla, 2014; Martin and Sospedra, 2014) and have been shown to influence cell proliferation and migration, cell differentiation and survival as well as neurite outgrowth and neurogenesis (Toman et al., 2004; Anderson and Maes, 2014; Blaho and Hla, 2014; Martin and Sospedra, 2014; Ye et al., 2016).

Several studies showed that these sphingolipid mediators and their enzymes are likely to have an integral role in different cell processes including proliferation, inflammation, apoptosis, and migration (Zheng et al., 2006; Maceyka et al., 2012).

S1P metabolism involves a number of different highly specialized enzymes. S1P is normally synthesized by sphingosine kinase-1 and−2 (SPHK1 and 2) and degraded by sphingosine-1-phosphate phosphatase (SGPP) or lyase (SGPL1) (see Figure 1) (Le Stunff et al., 2002; Morozov et al., 2013). SPHK1 activity is mainly associated with cell survival (Le Stunff et al., 2002; Morozov et al., 2013; Di Pardo et al., 2017a), while SPHK2 is widely described as being a dual-function protein whose activity may either guarantee the proper occurrence of physiological events like mitochondrial function and homeostasis as well as regulation of gene expression through inhibition of Class I HDACs or result detrimental mainly suppressing cell growth and promoting apoptosis (Maceyka et al., 2005; Hait et al., 2009; Riccio, 2010; Gomez et al., 2011; Strub et al., 2011).

On the other hand, the degradative enzymes SGPP and SGPL1 represent key regulators for the maintenance of balanced S1P levels and other sphingolipid intermediates that may control cell growth, proliferation and death (Serra and Saba, 2010). Uncontrolled up-regulation of SGPL1 typically results in a reduction of S1P availability and accumulation of hexadecenal, a biochemical condition previously reported to be cytotoxic (Kumar et al., 2011; Maceyka et al., 2012) that may likely contribute to neurodegenerative processes.

This mini-review will shortly highlight the current knowledge of research that now supports the idea that sphingolipids are intimately involved in disease progression and, together with altered expression/activity of metabolizing enzymes and associated receptors, can provide effective drug targets for the treatment of pathological states.

### Alteration of sphingolipid metabolism in neurodegenerative disorders

A fine balance between synthesis of sphingolipids and their degradation is normally required for many biological processes (Gault et al., 2010; Mullen et al., 2012), thus changes in their metabolism may profoundly affect brain homeostasis and function. Over the past few years, perturbed metabolism of the interconvertible bioactive sphingolipids, ceramide and S1P is increasingly becoming recognized as potential pathogenic factor in different neurodegenerative disorders (Table 1). Indeed, a plethora of new information identifying the importance of sphingolipids and their signaling pathways in these conditions has accumulated.

**Table 1:** Molecular alterations of sphingolipid metabolism detected in neurodegenerative disorders.

**Molecule**	**Alzheimer Disease**	**Huntington Disease**	**Parkinson Disease**
CERS2	Downregulated (Couttas et al., 2016)	Not Available	Not Available
Ceramides	Increased levels (Han et al., 2002; Cutler et al., 2004; Lee et al., 2004; Dinkins et al., 2015)	Increased levels (Pirhaji et al., 2016, 2017; Di Pardo et al., 2017a)	Increased levels (Ferrazza et al., 2016)
SPHK1	Downregulated (Ceccom et al., 2014b; Couttas et al., 2014)	Downregulated (Di Pardo et al., 2017a)	Downregulated (Sivasubramanian and Tay Ssw, 2013; Pyszko and Strosznajder, 2014)
SPHK2	Upregulated (Takasugi et al., 2011)	Upregulated (Di Pardo et al., 2017a; Moruno-Manchon et al., 2017)	Downregulated (Sivasubramanian and Tay Ssw, 2013; Pyszko and Strosznajder, 2014; Sivasubramanian et al., 2015)
SGPL1	Upregulated (Ceccom et al., 2014b)	Upregulated (Di Pardo et al., 2017a; Pirhaji et al., 2017)	Not Available
S1P	Reduced levels (Couttas et al., 2014)	Reduced levels (Pirhaji et al., 2016, 2017; Di Pardo et al., 2017a)	Not Available

#### Alzheimer's disease

Alzheimer's disease (AD) is the most common neurodegenerative disease and the leading cause of dementia in elderly people (Kumar et al., 2015). Progressive neurodegeneration in brain regions involved in learning and memory results in cognitive decline, loss of memory and changes of social and emotional behavior (Robins Wahlin and Byrne, 2011; Sona et al., 2013; Levenson et al., 2014). AD is characterized by extracellular accumulation of amyloid β-peptide (Aβ) toxic aggregates and intracellular deposits of abnormally phosphorylated tau protein (Blennow et al., 2006; Gouras et al., 2010).

A number of evidence demonstrates a key role of aberrant sphingolipid metabolism in the pathogenesis of the disease (Chakrabarti et al., 2016). Activity of ceramide synthetic enzyme 2 (CERS2), normally involved in the formation of long chain ceramide species (Levy and Futerman, 2010), has been recently found to be reduced in multiple brain regions of subjects at the preclinical stage of the disease (Couttas et al., 2016). Conversely, expression of genes involved in the *de novo* synthesis of sphingolipids is upregulated early in the disease progression (Katsel et al., 2007). Consistently, accumulation of ceramide has been reported in brain tissues from AD patients even at early stages of disease (Han et al., 2002; Cutler et al., 2004; He et al., 2010; Filippov et al., 2012) and may contribute to neurotoxic action of Aβ (Lee et al., 2004; Dinkins et al., 2015).

A number of evidence also indicates that, along with ceramide abnormalities, metabolism of other sphingolipids is affected in AD (Zheng et al., 2006; Mielke and Lyketsos, 2010). Also, alteration in the expression and/or in the activity of S1P-metabolizing enzymes as well as reduced levels of S1P has been widely reported in AD human brains (Katsel et al., 2007; Ceccom et al., 2014a,b; Couttas et al., 2014).

In particular, loss of SPHK1 and reduced bioavailability of S1P have been found early in the pathogenesis of the disease even before clinical diagnosis (Couttas et al., 2014). Conversely, upregulation of SPHK2 has been described to modulate Aβ release (Takasugi et al., 2011). Further indication of global derangement of sphingolipid metabolism in AD comes from the evidence of concomitant reduction in the levels of SPHK1 and S1P receptor 1 with enhanced expression of SGPL1 in post-mortem brains from human patients (Ceccom et al., 2014b).

#### Huntington's disease

Huntington's disease (HD) is the most common dominantly inherited brain disorder, characterized by progressive striatal and cortical neurodegeneration which results in motor, cognitive and behavioral disturbance (Mccolgan and Tabrizi, 2018). The disease derives from the expansion of a polyglutamine stretch (polyQ) (> 36 repeats) in the N-terminal region of the protein huntingtin (Htt) (Jimenez-Sanchez et al., 2017). Although the function of this protein is not completely known, expansion of the polyQ stretch endows mutant Htt (mHtt) with toxic properties, resulting in the development of a number of deleterious effects in both neuronal and non-neuronal cells (Maglione et al., 2005, 2006a,b; Carroll et al., 2015; Jimenez-Sanchez et al., 2017).

Among all cellular dysfunctions and biochemical defects, classically associated with the disease, defective metabolism of sphingolipids seems to play a key role in its pathogenesis (Di Pardo et al., 2017a).

Expression of S1P-metabolizing enzymes was reported to be aberrant in multiple HD settings (Di Pardo et al., 2017a,b; Pirhaji et al., 2017). Levels of SPHK1 was found reduced in brain tissues form two fully manifest HD mouse models (R6/2 and YAC128 mice), and most importantly, in brain tissues from HD patients (Di Pardo et al., 2017a). Conversely, levels of SGPL1 were increased in the brain of multiple disease animal models and in early manifest R6/2 mice (Di Pardo et al., 2017a), indicating that defect in sphingolipid metabolism occurs early in the disease stage likely contributing to its pathogenic process. First signs of early defective sphingolipid metabolism in HD have been reported also (Di Pardo et al., 2017a; Pirhaji et al., 2017) and such further evidence corroborates the hypothesis that similar alterations may conceivably contribute to the pathogenesis of the disease.

The imbalance in S1P-metabolizing enzymes results in decreased bioavailability of S1P and increased levels of ceramide species as reported in HD models (Pirhaji et al., 2016, 2017; Di Pardo et al., 2017a).

Ultimately, synthesis of *de novo* sphingolipids is also affected in HD animals, even at early stage of the disease (Di Pardo et al., 2017b). This alteration determines a robust reduction of certain dihydroceramide species along with dihydrosphingosine and dihydroS1P (Di Pardo et al., 2017b).

#### Parkinson's disease

Parkinson's disease (PD) is a neurodegenerative movement disorder with a prevalence of approximately 1 to 2% of the population over 65 years which increases up to 5% in people over 85 years old (Fahn, 2003; Obeso et al., 2010).

The pathological hallmarks of PD include the loss of dopaminergic neurons in the *substantia nigra pars compacta* and the formation of Lewy bodies mainly composed of aggregated alpha-synuclein (a-syn) protein and other components, including lipids (Gai et al., 2000; Halliday et al., 2005; Dickson et al., 2009).

Several studies demonstrated that defective ceramide metabolism may contribute to the pathogenesis of PD (Bras et al., 2008; Haughey, 2010; Fabelo et al., 2011). Mutations in the gene encoding for Glucocerebrosidase (GBA), a lysosomal enzyme converting glucosyl-ceramides into ceramide (see also Figure 1), increase the risk of developing sporadic PD (O'Regan et al., 2017).

Alterations in the expression of ceramide synthase genes as well as in the levels of certain ceramide species have been reported in post-mortem brain tissues from sporadic PD patients (Abbott et al., 2014). Consistently, mice knock out for Leucine-rich repeat kinase 2 (LRRK2), whose gene mutations cause inherited PD (Li et al., 2014), show a significant increase in the content of brain ceramide (Ferrazza et al., 2016).

Although very little, defects in S1P metabolism have been also reported in PD. Levels of SPHK1 and 2 have been described aberrant in both *in-vitro* and *in-vivo* models highlighting a potential contribution of S1P metabolism to the pathogenesis of the disease (Sivasubramanian and Tay Ssw, 2013; Sivasubramanian et al., 2015). Consistently, further studies demonstrate altered expression of S1P metabolizing enzymes with reduced activity for both SPHK1 and 2 in an *in-vitro* PD model (neuronal dopaminergic SH-SY5Y cells) induced by 1-methyl-4-phenylpyridinium (MPP+) (Pyszko and Strosznajder, 2014).

### Sphingolipid pathways as target for the development of potential therapeutic interventions

The study of bioactive sphingolipids as novel therapeutic targets for the treatment of brain disorders is still relatively young, however efforts are being made to question how therapeutics may be designed to take advantage of sphingolipid signaling pathways. Over the past few years it has been established that modulation of sphingolipid metabolism can lead to improved efficacy in many brain disorders ranging from neurodevelopmental to neurodegenerative settings (Deogracias et al., 2012; Asle-Rousta et al., 2013a; Di Pardo et al., 2014; Miguez et al., 2015; Moruno Manchon et al., 2015; Pirhaji et al., 2017; Zhao et al., 2017).

Cell survival and proper functioning of neuronal circuits is critical to the effectiveness of therapies in brain disorders. Sphingolipids are key signaling molecules regulating many of these cellular processes, thus it is very likely that manipulation of sphingolipid metabolism may represent a great way to develop more effective and targeted therapeutic strategies.

#### Alzheimer's disease

Very little is still known about the therapeutic effect that the modulation of sphingolipid metabolism may have in AD, however there is evidence demonstrating that stimulation of S1P receptors is beneficial in pre-clinical models of the diseases (Asle-Rousta et al., 2013a,b, 2014; Hemmati et al., 2013; Takasugi et al., 2013). Infusion of FTY720 (fingolimod), a non-selective S1P receptor modulator and FDA- approved drug for the treatment of Multiple Sclerosis, prevents spatial learning and memory impairments as well as Aβ induced-changes in the expression of some pro-apoptotic and inflammatory markers in AD animal models (Asle-Rousta et al., 2013a, 2014; Hemmati et al., 2013). Consistently, treatment with fingolimod leads to reduction of Aβ species in both *in-vitro* and *in-vivo* disease models (Takasugi et al., 2013).

Activation of specific S1P receptors also exerts beneficial effect in animal models of the disease. Indeed, administration of SEW2871, a S1PR_1_ selective agonist, ameliorates spatial memory impairments and attenuates the Aβ1-induced hippocampal neuronal loss in an AD rat model (Asle-Rousta et al., 2013b).

#### Huntington's disease

The effective therapeutic potential of the modulation of both levels and activity of S1P-metabolizing enzyme in HD is increasingly becoming evident in the last few years (Moruno Manchon et al., 2015; Di Pardo et al., 2017a; Pirhaji et al., 2017).

Recent evidence demonstrates that SPHK1 co-localizes with autophagosomes and its over expression favors autophagy-mediated clearance of mutant Htt exon-1 construct *in vitro* (Moruno Manchon et al., 2015). Coherently, stimulation of SPHK1, by the selective pharmacological activator K6PC-5 (Ji et al., 2015), significantly reduces apoptosis in mouse striatal derived HD cell lines and leads to the activation of pro-survival signaling pathways (Di Pardo et al., 2017a). The potential therapeutic validity of this pharmacological approach in HD is further supported by the beneficial effects that modulation of the kinase has in human HD iPSC-derived neurons (Di Pardo et al., 2017a).

Inhibition of SPHK2 or SGPL1 also represents a possible pharmacological strategy to increase survival in HD cell models (Di Pardo et al., 2017a; Moruno-Manchon et al., 2017; Pirhaji et al., 2017). Stimulation of S1P receptors is an additional therapeutically effective approach in this context. It stimulates the production of neurotrophins, activates neuronal pro-survival pathways and ultimately delays disease progression in HD mouse models (Di Pardo et al., 2014; Miguez et al., 2015).

#### Parkinson's disease

Modulation of S1P pathways has been recently explored in experimental models of PD. Pharmacological inhibition of SPHK1 reduces cell survival and increases oxygen reactive species in MPP+ human dopaminergic neuronal cells (Pyszko and Strosznajder, 2014). Conversely, treatment with S1P significantly reduced apoptosis in the same experimental model by regulating the expression of S1PR_1_ (Pyszko and Strosznajder, 2014).

Coherently, inhibition of SPHK2 leads to downregulation of mitochondrial-related genes such as proliferator-activated receptor γ coactivator-1α (PGC-1α) and its downstream targets nuclear respiratory factor 1 (NRF-1) and mitochondrial transcription factor A (TFAM) in multiple PD experimental models (Sivasubramanian et al., 2015). Furthermore, treatment with S1P enhance the expression of PGC-1α and NRF-1 in a mouse model of the disease and exert neuroprotective effect of dopaminergic neurons via S1PR_1_ (Sivasubramanian et al., 2015).

The potential role of S1PR_1_ stimulation in PD has been further explored. Treatment with FTY720 attenuates motor deficit and prevent dopaminergic neuronal loss in two chemical-induced PD models (Zhao et al., 2017). The neuroprotective effect of the drug is associated with the activation of pro-survival kinase ERK in both *in-vitro* and *in-vivo* PD models (Zhao et al., 2017). The benefit of FTY720 was abolished by the co-treatment with W146, a S1PR_1_ selective antagonist, indicating that action of fingolimod was mediated, at least in part, by its binding at S1PR_1_.

## Concluding remarks and future perspectives

In the last few years, the recognition of aberrant sphingolipid metabolism is becoming more evident in neurodegenerative disorders and, its deeper investigation may either strongly contribute to a better understanding of the disease pathogenesis or support the development of novel and more targeted therapeutic approaches.

Defects in this metabolism may profoundly affect the CNS and may interfere with selective biological pathways, whose dysregulation can explain some of the molecular and cellular alterations underlying neurodegeneration.

The hypothesis of candidating sphingolipid pathways as attractive therapeutic targets is strongly supported by evidence that demonstrates that modulation of such pathways has beneficial effects in different neurodegenerative conditions. To date, what makes sphingolipid metabolism a promising target with a real potential to be successfully translated into clinical practice of brain disorders is the evidence that some of the drugs targeting S1P and its receptors are already marketed or in advanced phases of clinical development for the treatment of human disorders (Hatoum et al., 2017; O'Sullivan and Dev, 2017). This could certainly allow to take advantage from the already available molecules and eventually to promote the development of new ones.

Takin into account the diverse functions that some sphingolipids may have, reducing ceramide accumulation and/or targeting its conversion into other sphingolipid species may represent an alternative approach for brain disorders (Brodowicz et al., 2018). In this context, the feasibility of lowering ceramide levels and its effectiveness is supported by a number of pre-clinical studies and by the evidence that some drugs that target ceramide production are currently approved for clinical use (Kornhuber et al., 2010). On the other hand, boosting ceramide metabolism toward the synthesis of C1P and/or S1P, through the use of specific kinase activators (Kwon et al., 2007; Tada et al., 2010), may also represent a fascinating approach.

## Author contributions

AD and VM co-wrote the manuscript.

### Conflict of interest statement

The authors declare that the research was conducted in the absence of any commercial or financial relationships that could be construed as a potential conflict of interest.
